# Characterization of a Nonclassical Class I MHC Gene in a Reptile, the Galápagos Marine Iguana (*Amblyrhynchus cristatus*)

**DOI:** 10.1371/journal.pone.0002859

**Published:** 2008-08-06

**Authors:** Scott Glaberman, Louis Du Pasquier, Adalgisa Caccone

**Affiliations:** 1 Department of Ecology and Evolutionary Biology and the Yale Institute for Biospheric Studies, Yale University, New Haven, Connecticut, United States of America; 2 Institute of Zoology and Evolutionary Biology, University of Basel, Basel, Switzerland; American Museum of Natural History, United States of America

## Abstract

Squamates are a diverse order of vertebrates, representing more than 7,000 species. Yet, descriptions of full-length major histocompatibility complex (MHC) genes in this group are nearly absent from the literature, while the number of MHC studies continues to rise in other vertebrate taxa. The lack of basic information about MHC organization in squamates inhibits investigation into the relationship between MHC polymorphism and disease, and leaves a large taxonomic gap in our understanding of amniote MHC evolution. Here, we use both cDNA and genomic sequence data to characterize a class I MHC gene (*Amcr-UA*) from the Galápagos marine iguana, a member of the squamate subfamily Iguaninae. *Amcr-UA* appears to be functional since it is expressed in the blood and contains many of the conserved peptide-binding residues that are found in classical class I genes of other vertebrates. In addition, comparison of *Amcr-UA* to homologous sequences from other iguanine species shows that the antigen-binding portion of this gene is under purifying selection, rather than balancing selection, and therefore may have a conserved function. A striking feature of *Amcr-UA* is that both the cDNA and genomic sequences lack the transmembrane and cytoplasmic domains that are necessary to anchor the class I receptor molecule into the cell membrane, suggesting that the product of this gene is secreted and consequently not involved in classical class I antigen-presentation. The truncated and conserved character of *Amcr-UA* lead us to define it as a nonclassical gene that is related to the few available squamate class I sequences. However, phylogenetic analysis placed *Amcr-UA* in a basal position relative to other published classical MHC genes from squamates, suggesting that this gene diverged near the beginning of squamate diversification.

## Introduction

Class I major histocompatibility complex (MHC) molecules are well known for their pivotal role in the recognition of altered self cells (e.g. virus-infected cells) by T cytotoxic (T_C_) cells. In this process, short peptide fragments derived from pathogens within the host cell are bound to class I receptor structures and transported to the cell surface. Here, the paired receptor/antigen complex is recognized by CD8^+^ T_C_ cells, initiating a sequence of events that ultimately leads to the lysis of the infected host cell [1], [2].

Since class I antigen presentation is essential for the cell-mediated clearance of intracellular pathogens, it is not surprising that these molecules are expressed on most somatic cells and in the majority of host tissue types [3]. An additional characteristic of the genes encoding class I receptors is a high level of polymorphism, which enables the host to recognize and bind a wide array of foreign peptides [4]. However, as the number of descriptions of vertebrate MHC loci has increased, it has become clear that many class I genes do not possess the features described above.

The classification of vertebrate class I loci has been divided into two general categories: classical (or class Ia) and nonclassical (or class Ib). Classical genes are those found within the MHC region, possessing high polymorphism, strong and wide expression, and are involved in the presentation of endogenous antigens to T_C_ cells. Classical loci have been well characterized in humans (HLA-A, -B, and -C) and mice (H-2K, D, and L) but are also well described in some other mammals and in fish, and to a lesser to degree in amphibians, birds, and non-avian reptiles.

Nonclassical genes can be located inside or outside of the MHC and typically possess little or no polymorphism and weak expression that is often limited to specific tissue types. Class Ib molecules are known to perform a diverse array of functions which include the recognition of antigenic lipids (human and mouse CD1) [5], the binding and transportation of classical class I molecules within the host cell (human HLA-E and mouse Qa-1^b^) [6], and the targeting of evolutionarily conserved protein epitopes of pathogens (mouse H2-M3) [7]. There are even nonclassical loci whose functions are not related to immunity. For example, the human HFE molecule is known to play an important role in iron metabolism [6].

Phylogenetic studies depict an extremely complex evolutionary history of class I genes, where both classical and nonclassical loci exhibit little orthology across mammals as well as among vertebrates in general [8]. Since particular species, or closely related taxa, often possess exclusive sets of paralogous genes, it appears that class I lineages have undergone repeated, independent expansion and diversification events over the course of vertebrate evolution. This pattern has been shown to be congruent with a birth-and-death model of evolution, where loci are frequently duplicated and lost, even over short timescales [9]. However, concerted evolution is also thought to contribute to the close relationship of class I genes within species by the homogenization of even divergent lineages through inter-locus gene conversion [10], [11]. Although the function of most nonclassical genes is not well understood, their presence within these independently expanded class I clades is a testament to their importance in vertebrate immunity.

Since class I evolution is known to be highly erratic, large taxonomic gaps in MHC characterization prevent the examination of the events and processes which have led to class I diversification. Therefore, a comparative phylogenetic approach may serve as an important tool for understanding class I history. This was recently demonstrated in the gray short-tailed opossum (*Monodelphis domestica*) where 11 class I loci were shown to have diverged after the split of marsupials from other mammals, yielding insight into the timing and breadth of class I differentiation within this lineage [12].

Among vertebrates, non-avian reptiles are the most poorly represented taxon in terms of MHC data. For example, the order Squamata, which includes lizards and snakes, contains over 7,000 species that have been independently evolving for over 200 million years [13]–[15]. Yet, only a single study characterizing full-length class I genes has been published in the last 15 years [16]. Squamates, together with the only two extant species in the order Sphenodontia, make up the superorder Lepidosauria, which is the other main lineage of amniotes in addition to archosaurs (birds and crocodilians) and mammals. Given the vast differences in the organization of mammalian and avian MHC regions [11], [17], the description of squamate class I genes can therefore provide a firmer phylogenetic basis upon which to reconstruct the characteristics of the ancestral amniote MHC, an important launching point for understanding how the mode of MHC evolution differs among major vertebrate lineages.

In this study, we use both cDNA and genomic sequence data to characterize a class I gene from the Galápagos marine iguana (*Amblyrhynchus cristatus*), a member of the squamate subfamily Iguaninae. We also present partial fragments of a similar gene from other iguanine species. The marine iguana sequence possesses several hallmarks of a nonclassical locus and can serve as the basis for more detailed functional studies of class I genes in this group. We compare this sequence to the few others available from squamates, including those from a recent study of class Ia genes from marine iguanas and two other iguanines, the Galápagos land iguana (*Conolophus subcristatus*) and the common green iguana (*Iguana iguana*) [16]. The information provided here will hopefully aid in the collection of additional class I data from squamates and fill in the vast phylogenetic gap that is missing in our understanding of amniote MHC evolution.

## Materials and Methods

### RNA isolation and cDNA synthesis

Total RNA was isolated from the blood of a single marine iguana from the island of Santa Cruz, Galápagos, using the Tri Reagent BD kit (Molecular Research Center, Cincinnati, OH, USA) and the protocol provided. Complementary DNA (cDNA) was synthesized using the SuperScript III RT enzyme and reagents provided in the GeneRacer kit (Invitrogen, Carlsbad, CA, USA). The complete kit protocol was followed for generating first-strand cDNA pools with intact 5′ ends except that both random hexamers and the oligo dT primer provided in the kit were used in a single reverse transcription reaction to obtain full-length cDNA fragments.

### 5′ and 3′ RACE of marine iguana cDNA

**Figure 1:**
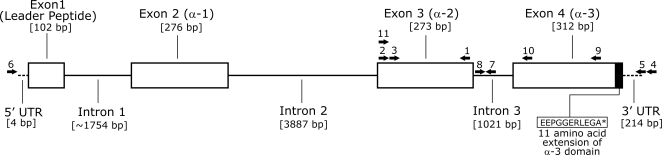
Map of the *Amcr-UA* class I gene based on genomic and cDNA data. All exons are scaled relative to each other. Introns are also scaled relative to each other, but are more condensed than exons. Asterisk in the α-3 amino acid sequence represents the termination signal. Numbers above arrows show the position of primers used in this study and correspond to the numbering scheme in Table 1 – except for primer 11, which has been published elsewhere [18].

In order to design gene-specific primers for 3′ RACE, a small fragment (201 base pairs [bp]) in the class I α-2 domain was amplified by PCR using a forward primer described in Radtkey et al. [18] and a degenerate primer (MHCI-R3) designed from an alignment of a range of vertebrate class I sequences (see Table 1 for primer list and sequences; see Figure 1 for primer locations). Sequences derived from the small, amplified fragment were used to design two forward-facing gene-specific primers for use in 3′ RACE (IgNC-F1 and IgNC-F2).

**Table 1:** Gene-specific primers used in this study.

Primer Name	Sequence (5′ to 3′)	Target Region
(1) MHCI-R3	ASGTAYYTCBBCAGCCACT	Initial exon 3 genomic amplification
(2) IgNC-F1	TGYGAGCTGAGGAAAGATGGGAGCATAG	3′ RACE
(3) IgNC-F2	TTACCARTGTGCTTATGATGGGAGGGAC	3′ RACE (Nested)
(4) MHC17H3-5R-R1	TTCATGCAGTCCAAAGGCAGCAG	5′ RACE; Genomic fragment
(5) MHC17H3-5R-R2	TCTTCTGCCTTGCTTCTGTCAAATATGGAG	5′ RACE (Nested)
(6) MHC1-Gr2-F1	ACCGAGAGGGTTGAGCTGGAGAG	Genomic fragment
(7) MHCI-Int-R1	TGAGGCTGASAGAGTGTAATCCTCCC	Iguaninae exon3/intron3 fragment
(8) MHC1-Int2-F2	AGRTGTRTGTAATATATCATCCAGG	Intron3/exon4 fragment (All Iguaninae)
(9) MHC1-R4	CAGRCYGGYRTGMTCCACRCGSCAC	Intron3/exon4 fragment (*Amcr; Cosu*)
(10) MHC1-R6	TCTCCTTGGGGTAGAAGCCRTC	Intron3/exon4 fragment (*Cyco*; *Ctde*; *Cyca*; *Ctcl*; *Cyri*)

Numbers before primer refer to location in the gene as indicated by arrows in Figure 1.

Footnote: See Materials and methods for Iguaninae taxa abbreviations.

For the 3′ RACE, nested PCR was performed on marine iguana first-strand cDNA. The first round of PCR utilized the oligo dT adaptor-specific primer provided in the GeneRacer kit and the primer Ig-NC-F1. The PCR was performed with the following reagents and concentrations: 1U Phusion High Fidelity DNA Polymerase (Finnzymes, Espoo, Finland), 200 µM of each dNTP, 0.5 µM gene-specific primer, 1× Phusion HF Buffer with MgCl_2_. Because the melting temperature of the gene-specific primer was >72°C, a two-step PCR was carried out with an initial denaturation (denat) of 98°C for 30 s followed by 30 cycles of 98°C for 10 s and 72°C for 60 s, and a final extension (ext) of 72°C for 10 min. Nested 3′ RACE was carried out under the same conditions as the initial PCR using the gene-specific primer Ig-NC-F2, the nested oligo dT adaptor primer provided, and 1 µl of PCR product from the initial 3′ RACE.

Sequence data derived from 3′ RACE products were then used to design two gene-specific reverse primers (MHC17H3-5R-R1 and MHC17H3-5R-R2) in the 3′ UTR for use in 5′ RACE. In the first round of PCR, an adaptor-specific primer provided by the kit was used in combination with the gene-specific primer MHC17H3-5R-R1. PCR was performed in a 50 µl reaction with the following reagents and concentrations: 2.5 U Platinum *Taq* DNA Polymerase High Fidelity (Invitrogen), 200 µM of each dNTP, 0.2 µM gene-specific primer, 2 mM MgSO_4_, 1× High Fidelity PCR Buffer. Cycling conditions were as follows: initial denat at 94°C for 2 min followed by 5 cycles of 94°C for 30 s and 72°C for 2 min; 5 cycles of 94°C for 30 s and 70°C for 2 min; 23 cycles of 94°C for 30 s, 59°C for 30 s, and 68°C for 2 min; and a final ext for 10 min at 68°C. The nested 5′ RACE step was carried out with the nested 5′ adaptor primer and the primer MHC17H3-5R-R2 under the following conditions: initial denat at 94°C for 2 min; 25 cycles with 30 s of denat at 94°C, 30 s of annealing at 61°C, 2 min ext at 68°C; and a final ext of 68°C for 20 min.

The nested RACE products from both the 5′ and 3′ procedures were ligated into the pCR4-TOPO vector, transformed, and grown overnight on LB plates following the manufacturer's protocol for the TOPO TA Cloning Kit for Sequencing (Invitrogen). Approximately ten clones each from 5′ and 3′ RACE were added to 25 µl of water and heated at 94°C for 10 min to lyse cells. 1 µl of this solution was added to a 25 µl PCR reaction containing 1.5 U AmpliTaq Gold DNA Polymerase (Applied Biosystems, Foster City, CA, USA), 200 µM of each dNTP, 1 µM standard T3 and T7 primers, 1.5 mM MgCl_2_, and 1× PCR Buffer without MgCl_2_. Cycling conditions were as follows: initial denat at 94°C for 10 min, followed by 30 cycles with 30 s of denat at 94°C, 30 s of annealing at 55°C, and 90 s ext at 72°C; and a final ext of 72°C for 10 min. PCR products were purified by filtration using either the QIAquick PCR purification kit (Qiagen; Valencia, CA, USA) or Millipore (Billerica, MA, USA) filter plate and sequenced using a 3730 DNA Analyzer (Applied Biosystems).

### PCR amplification of Amcr-UA from genomic DNA

RACE products yielded a complete coding class I sequence designated *Amcr-UA*. (see Results). In order to amplify this gene from genomic DNA, total DNA was extracted from the whole blood of a single marine iguana using standard phenol-chloroform extraction [19]. Primers designed in the 5′ and 3′ UTR of the marine iguana cDNA sequence were used to amplify the corresponding genomic fragment from the total DNA (Table 1). This individual was not the same as the one used in RNA extraction and RACE but did come from the same population (Santa Cruz) and is likely to be closely related. PCR conditions were similar to those used in 3′ RACE with the Phusion enzyme, except that the two-step ext time was increased to 4 min. Due to the large size of the resultant fragment (∼8 kb), numerous internal primers (available upon request) had to be designed in addition to the original PCR primers in order to obtain the complete sequence.

### Population-level data of Amcr-UA

PCR targeting a portion of exon 3, which corresponds to the α-2 domain, and intron 3 was performed on genomic DNA from 65 marine iguanas representing three different islands in the Galápagos archipelago: Santa Cruz, N = 31; Isabela, N = 18; Fernandina, N = 16. Exon 3 was targeted because it is known to be highly variable in class Ia genes and contains sites that are involved in antigen recognition [20]. Therefore, we felt that the presence or absence of nucleotide variation here would be satisfactory for determining whether this gene is polymorphic or not. The forward primer described in Radtkey et al. [18] was used in combination with the primer MHCI-Int-R1 in a 25 µl PCR reaction with the following reagents and conditions: 2.5 U AmpliTaq DNA Polymerase (Applied Biosystems), 200 µM of each dNTP, 1 µM of each primer, 1.5 mM MgCl_2_, and 1× PCR Buffer without MgCl_2_. Cycling conditions were as follows: initial denat at 94°C for 5 min, followed by 33 cycles with 30 s of denat at 94°C, 30 s of annealing at 59°C, and 60 s ext at 72°C; and a final ext of 72°C for 10 min. PCR products were purified and sequenced as described above.

### Iguaninae species data collection

A genomic fragment homologous to the *Amcr-UA* sequence was targeted in six other species in the same subfamily as *Amblyrhynchus* (Iguaninae): *Conolophus subcristatus*, *Cyclura carinata*, *Cyclura cornuta*, *Cyclura rileyi*, *Ctenosaura defensor*, and *Ctenosaura clarki* (for all table and figures, taxa are labeled with the following abbreviations: *Amcr = Amblyrhynchus cristatus; Cosu = Conolophus subcristatus; Cyco = Cyclura cornuta; Cyca = Cyclura carinata; Cyri = Cyclura rileyi; Ctde = Ctenosaura defensor; Ctcl = Ctenosaura clarki*). This fragment corresponds to the majority of exon 3, the entire intron 3, and a small portion of exon 4 in the *Amcr-UA* sequence, and was amplified in two separate but overlapping fragments. The first fragment, which spans exon 3 and the first part of intron 3, is identical to the one amplified in the population sample of *Amblyrhynchus*, and was generated using the same PCR protocol described above. The second fragment covers the second part of intron 3 and the very beginning of exon 4. The forward primer used to amplify this fragment was the same for all taxa (MHC1-Int-F2), but a different reverse primer (MHC1-R4) was used to amplify *Amblyrhynchus* and *Conolophus* specimens than for the other species (MHC1-R6). The PCR reagent concentrations for both primer pairs were the same as for the exon 3/intron 3 fragment, but the cycling conditions for the MHC1-Int-F2/MHC1-R4 primer combination were as follows: initial denat at 94°C for 5 min, followed by 35 cycles with 40 s of denat at 94°C, 40 s of annealing at 60°C, and 2 min 30 s ext at 72°C; and a final ext of 72°C for 15 min. The PCR cycling conditions for the MHC1-Int-F2/MHC1-R6 primer combination were 94°C for 5 min for initial denat, followed by 35 cycles with 45 s of denat at 94°C, 45 s of annealing at 55°C, and 60 s ext at 72°C; and a final ext of 72°C for 10 min.

### Data analysis

Sequences from the *Amcr-UA* genomic fragment were aligned in the program SEQUENCHER 4.2.2 (Gene Codes Corporation, Ann Arbor, MI, USA). Comparison of the genomic and cDNA sequences was used to identify the exon/intron structure of *Amcr-UA*.

The program MUSCLE v3.6 [21] was used to produce full-length amino acid and exon 4 (α-3 domain) nucleotide alignments for *Amcr-UA* and the following vertebrate class I sequences from GenBank: Galápagos marine iguana (*A. cristatus*), Amcr-UB*01 EU604308, Amcr-UB*02 EU604309, Amcr-UB*03 EU604310, Amcr-UB*0401 EU604311, Amcr-UB*0402 EU604312; Galápagos land iguana (*C. subcristatus*), Cosu-UB*0101 EU604313, Cosu-UB*0102 EU604314, Cosu-UB*02 EU604315, Cosu-UB*03 EU604316; Green iguana (*Iguana iguana*), Igig-UB*0101 EU604317, Igig-UB*0102 EU604318, Igig-UB*02 EU604319; Ameiva lizard, *LC5* M81095, *LC25* M91097; Northern water snake (*Nerodia sipedon*), *SC1* M81099; Chinese soft-shelled turtle (*Pelodiscus sinensis*), AB185243; Chicken (*Gallus gallus*), *B-F10* X12780; Mallard (*Anas platyrhynchos*), *Du2* AB115242; Great reed warbler (*Acrocephalus arundinaceus*), *cN3* AJ005503; Axolotl (*Ambystoma mexicanum*), *Amme-3* U83137; African clawed frog (*Xenopus laevis*), *UAA-1f* L20733; Mouse (*Mus musculus*), *H2K* L36312, *H2-D1* NM_010380, *H2-Q1* NM_010390, *H2-Q10* NM_010391; Wallaby (*Macropus rufogriseus*), Maru-UB*01 L04952; Platypus (*Ornithorhynchus anatinus*), *Oran2-1* AY112715; Possum (*Trichosurus vulpecula*), *Trvu-UB* AF359509; Rainbow trout (*Oncorhynchus mykiss*), *Onmy-UBA* AF287487; Zebrafish (*Danio rerio*), *Dare-UBA* NM131471; Human, *HLA-B7* U29057, *HLA-Cw* D50852. This set of sequences is similar to the one used in a study of tuatara class I genes by Miller et al. [22] as well as the recent study of iguanine class Ia loci [16], and was chosen for consistency. For the protein data, percent identity between *Amcr-UA* and other vertebrate class I genes was derived from *p*-distance values calculated separately for each structural domain in the program MEGA 4.0 [23]. Conserved vertebrate class I amino acid positions were identified following Kaufman et al. [24].

The exon 4 alignment provided the basis for Bayesian, maximum likelihood (ML), and neighbor-joining (NJ) phylogenetic reconstruction. The program MRMODELTEST v2 [25], which is based on code from the MODELTEST software [26], was used to compare the fit of different nucleotide substitution models to the vertebrate dataset. The general time reversible model (GTR) with additional parameters for gamma distribution and fraction of invariable sites provided the best fit to the data according to both the hierarchical likelihood ratio test and the Akaike information criterion. This model was implemented in a Bayesian framework using the program MRBAYES [27] as well as in ML reconstruction using the TREEFINDER software [28]. For Bayesian analysis, the default software settings were used, and the search was run for 2,000,000 generations with the first 10% of parameter samples discarded as burn-in. In the ML analysis, 1,000 bootstrap replicates were run to assess support for specific nodes. NJ search and bootstrap analysis were carried out in PAUP using the GTR substitution model with 500 replicates.

Sequence fragments spanning the majority of exon 3 and all of intron 3 in the seven iguanine species were aligned in MEGA, and *p*-distance values were calculated separately for the exon and intron.

Sequences generated in this study were deposited in GenBank under the following accession numbers.: EU839663 (full-length *Amcr-UA* cDNA); EU839664 (*Amcr-UA* genomic fragment); EU839665-EU839670 (Iguaninae exon 3/intron 3).

## Results

### Characterization of cDNA sequences from *Amblyrhynchus cristatus*

**Figure 2:**
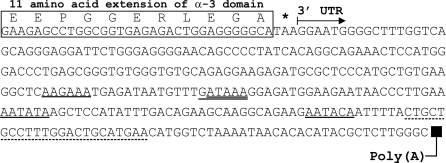
3′ sequence map of the *Amcr-UA* fragment generated from cDNA. Possible polyadenylation sites are labeled with a solid underline. The asterisk marks the termination signal at the end of exon 4 (α-3 domain). The eleven amino acid residues that mark the unusual extension of the α-3 domain are labeled above their respective coding nucleotide sequences. The sequence of primer MHC17H3-5R-R1 is labeled with a broken underline; this was the reverse primer used to generate the genomic DNA sequence and shows how far the genomic sequence stretches downstream relative to the cDNA.

Multiple clones were sequenced from PCR products generated by both 5′and 3′ RACE of marine iguana cDNA. For 3′ RACE, resulting clones carried two unique class I-like sequences. One of these became the subjected of another study [16], while the other was the focus of this paper, and was used to design specific 5′ RACE primers. The sequence of this 3′ RACE clone was identical in the area of overlap with the single class I fragment obtained from 5′ RACE. When aligned, these sequences comprised a 1,294 bp fragment which spanned the complete coding sequence (CDS) as well as the 5′ and 3′ UTRs of a single class I sequence type (Figure 1). This gene was labeled *Amcr-UA* based on established nomenclature rules [29]. Although the 3′ UTR sequence reaches the site of polyadenylation, neither of the canonical polyadenylation signals (AATAAA or ATTAAA) were found. However, there are several alternative motifs at a range of positions in the 3′ UTR that are known to be involved in polyadenylation, including AATACA, AATATA, GATAAA or ATAAA, and AAGAAA, which are located 64, 101, 131, and 150 bp upstream of the poly(A) region respectively (Figure 2; see [30]–[34] for characterization of alternative polyadenylation sites).

**Figure 3:**
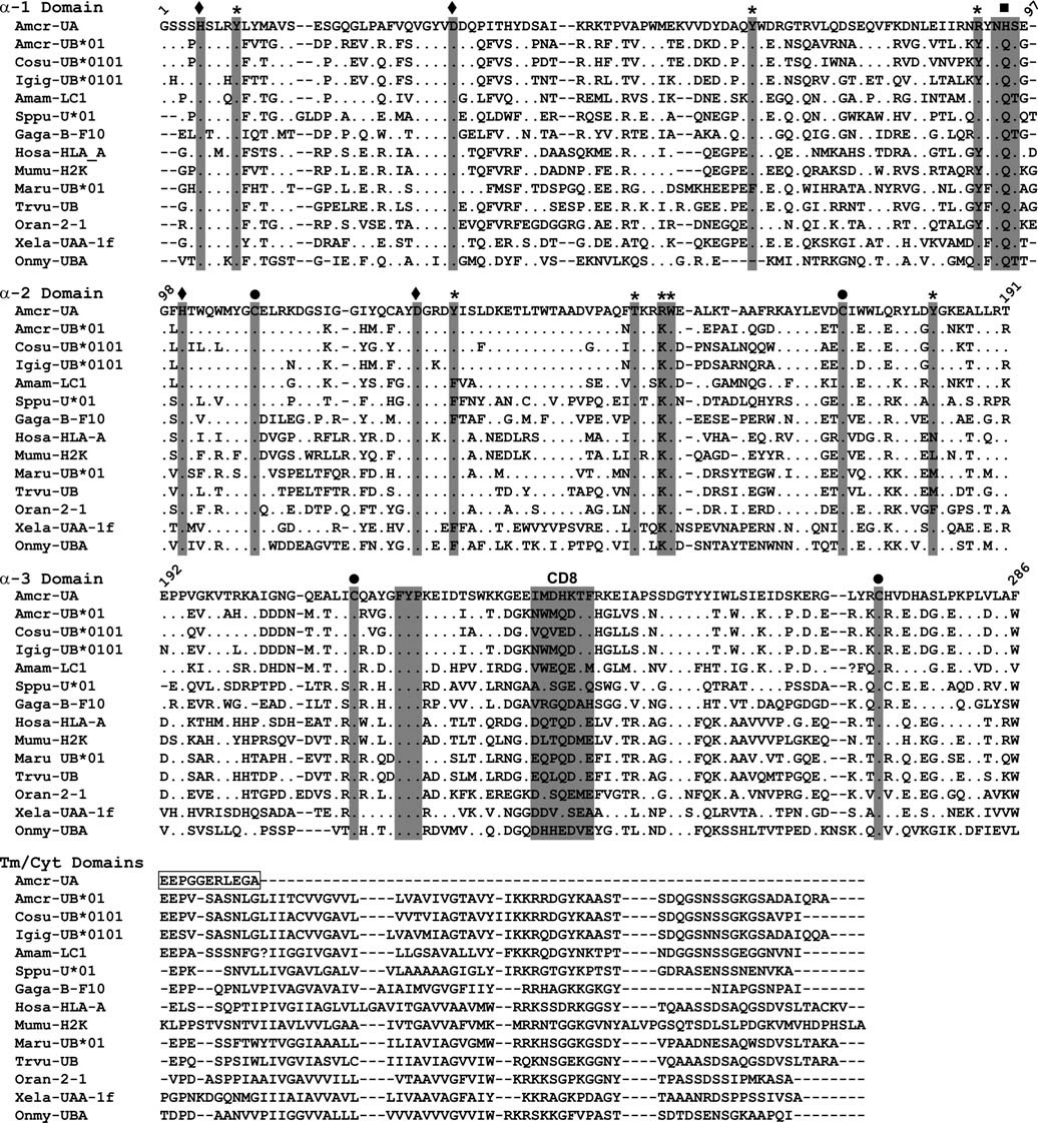
Amino acid alignment of *Amcr-UA* with other vertebrate class I sequences. Coding domains are separated according to Koller and Orr [52] and exon/intron information from the *Amcr-UA* genomic sequence. Numerical labels in the alignment refer to amino acid positions in the *Amcr-UA* sequence, not in the alignment itself. Shaded columns indicate amino acid positions that are conserved or have expected functions. These amino acid positions also contain the following additional labels: “asterisks” = conserved peptide-binding residues of antigen N- and C- termini; “diamonds” = salt bridge-forming residues; “circles” = disulfide bridge-forming cysteines; “squares” = N-glycosylation site; “CD8” = expected CD8 binding site. The boxed sequence from *Amcr-*UA represents the 11 amino acid extension that is encoded in exon 4, which corresponds to the α-3 domain. Information and accession numbers of other vertebrate class I sequences can be found in the methods section.

One of the most striking features of the *Amcr-UA* sequence is the absence of the transmembrane (Tm) and cytoplasmic (Cyt) domains that are characteristic of most classical and nonclassical class I MHC genes. The CDS extends only 11 amino acid residues downstream from the expected end of the α-3 domain. The first three of these residues (EEP) are identical to the first three Tm domain residues of the classical *UB* genes from the three iguanine species as well as the Ameiva lizard (*LC1*) (Figure 3); but the other eight residues have no clear homology to any of the other loci. There was no evidence of a longer 3′ RACE fragment that might contain a full-length version of the truncated *Amcr-UA* sequence.

While the structure and composition of class I genes are quite variable within and between vertebrate groups, the *Amcr*-*UA* sequence possesses many of the conserved amino acid residues that are characteristic of class I genes, and even some residues that are common in classical class I genes (indicated by asterisks in Figure 3). For example, there are nine class I α chain residues which are highly conserved across vertebrates and are known to be involved in the binding of N- and C-termini of antigenic peptides [24], [35], [36]. Seven of these residues are shared between the *Amcr-UA* sequence (positions in *Amcr-UA*: Y9, Y62, Y124, T144, W148, Y160, and Y175) and the common reference type found in the classical Human HLA-A, -B, and -C, molecules. Moreover, one of the two remaining positions (R87) matches the predominant character state in non-mammalian vertebrates, including lizard, fish, birds, tuatara, and amphibians. Several studies have used the presence of these conserved peptide-binding residues in different species to distinguish classical antigen-presenting class I genes from nonclassical class I genes with alternative functions [36]–[38].

Other features that are shared between the marine iguana sequence and nearly all class I genes of other vertebrates include four cysteine residues at positions 103, 165, 204, and 260, which are involved in intradomain disulfide bridge formation, two salt bridges at positions H5-D31 and H95-D120, and the highly conserved FYP motif at positions 209–211 in the α-3 domain. Class I genes of most species contain a NQS or NQT glycosylation site near the end of the α1 domain; however, the *Amcr-UA* sequence contains a histidine (H) rather than a glutamine (Q) at position 90, which is not the case in other vertebrates, including the classical sequences from the three iguanine species as well as the Ameiva lizard. But this residue is not known to be important for N-linked glycan formation. The amino acid residues which correspond to the CD8 binding site of the HLA-A locus are at positions 224–230 in the *Amcr-UA* fragment, but three of the seven residues are non-polar, which would be unexpected at these sites for a classical class I gene; however this region is known to be highly variable among species, and the coevolution of the CD8 ligand and its class I binding site is not well understood [24].

### Genomic sequence of *Amcr-UA*

Amplification of the *Amcr-UA* gene was carried out on marine iguana genomic DNA using specific primers designed in the 5′ and 3′ UTR of the cDNA sequence. The resulting PCR product was approximately 8 kb and was identical to the cDNA in all areas of overlap, except for a single nucleotide position in the expected signal peptide. Some degree of mismatch between the cDNA and genomic sequences was anticipated since these fragments were derived from different individuals. A small portion (<100 bp) of intron 1 in the amplified genomic fragment could not be sequenced from either direction after several attempts. Secondary structure analysis revealed that the ∼80 bp before and after the unsequenced region are highly complementary (∼80%) and could form a secondary loop. However, even after addition of DMSO, which is known to relax secondary structure during cycle sequencing, there was still a consistent and steep drop-off of chromatogram peaks in this region.

The structure of the genomic fragment is shown in Figure 1. This sequence revealed that the 11 amino acid residues (plus stop codon) that extend from the expected end of the α-3 domain are encoded in the same exon as the α-3 domain itself (Figure 1). In other vertebrates, separate exons usually code for the Tm/Cyt and α-3 domains. Yet the “EEP” amino acid motif, which marks the beginning of the Tm/Cyt domain in the classical class I sequences from the three iguanine species and the Ameiva lizard, is part of the α-3 encoding exon in *Amcr-UA*, and might be the result of some type of genomic rearrangement.

Besides the missing Tm/Cyt domains, the position of exons and introns are the same as in the majority of class I genes, where the signal peptide and each of the three extracellular domains (α-1, α-2, and α-3) are encoded by separate exons with intervening introns. The missing fragment from intron 1 was estimated to be ∼100 bp by comparing the band size to a DNA ladder on an agarose gel.

### Relationship of *Amcr-UA* to Class I sequences of other vertebrates

Over the three extracellular domains, the amino acid identity between the *Amcr-UA* sequence and other published vertebrate class I genes ranges from 33% in the Rainbow trout (*Oncorhynchus mykiss*) to between 56.2% and 60.0% for the iguanine *UB* sequences (Table 2). Considered separately, the α-2 and α-3 domains are most similar to the classical iguanine sequences, but the α-1 domain is approximately as close to the tuatara (45.5%–50.0%) and Ameiva lizard (46.7%) as it is to the iguanine *UB* sequences (43.5%–50%).

**Table 2:** Percent amino acid identities between *Amcr-UA* and class I sequences from other vertebrates.

Species	Percent Identity with *Amcr-UA*			
	α1	α2	α3	α1, α2, α3
Marine iguana (*Amcr-UB*)	43.5–46.7	74.7–75.8	52.2	56.2–57.8
Land iguana (*Cosu-UB*)	45.7–50.0	68.1–75.8	56.5	57.8–60.0
Green iguana (*Igig-UB*)	46.7–48.9	73.6–76.7	53.3	57.8–59.5
Ameiva Lizard (LC1)	46.7	64.8	48.4	53.3
Tuatara (*Sppu-U*)	45.5–50.0	50.5	36.3	44.1–45.6
Chicken (*Gaga*-B-F10)	40.9	52.2	36.3	43.1
Mallard (*Anpl*-Du2)	45.5	56.0	40.7	47.4
Warbler (*Acar*-cN3)	43.2	46.2	44.0	44.4
Axolotl (*Amme*-3)	43.2	54.9	34.8	44.4
Xenopus (*Xela*-UAA-1f)	44.3	44.0	37.4	41.9
Human (*Hosa*-HLA-A)	43.7	53.8	38.0	45.2
Mouse (*Mumu*-Q10)	44.8	57.8	34.8	45.7
Mouse (*Mumu*-H2K)	43.7	52.2	31.5	42.4
Wallaby (*Maru*-UB*01)	40.0	54.9	38.0	44.3
Platypus (*Oran*-2-1)	40.9	56.0	32.6	43.2
Possum (*Trvu*-UB)	46.7	56.0	34.8	45.8
Nurse Shark (*Gici*-UAA)	41.4	44.0	32.2	39.2
Rainbow Trout (*Onmy*-UBA)	33.3	40.7	24.7	33.0
Zebrafish (*Dare*-UBA)	27.6	51.6	24.7	34.8

Values were calculated for each extracellular domain separately and for all three domains combined.

**Figure 4:**
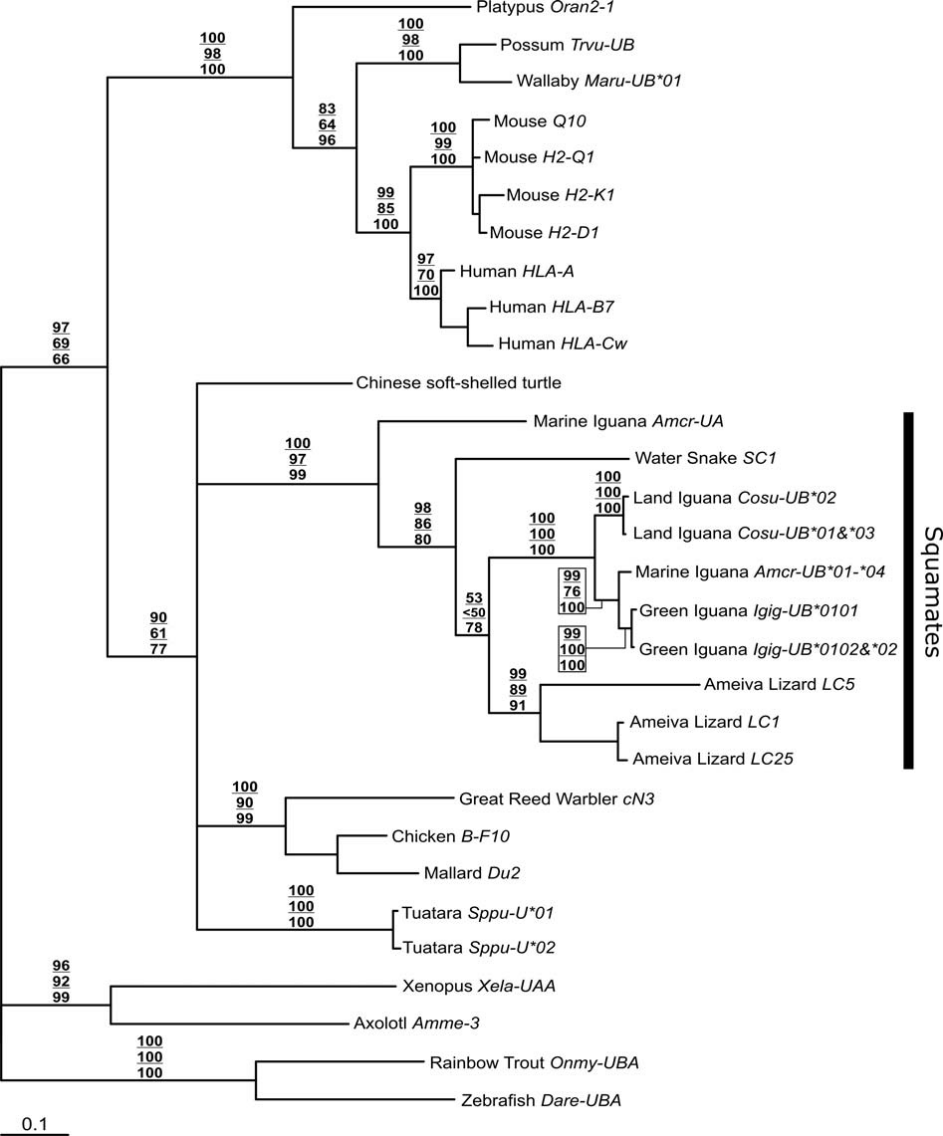
Class I phylogenetic reconstruction using α-3 (exon 4) sequence data from the major vertebrate groups. The top number at each node is the Bayesian posterior distribution value supporting the topology displayed. The middle and bottom numbers are bootstrap values from maximum likelihood and neighbor joining searches respectively. Branch lengths are represented as expected number of substitutions per site and were inferred along with topology in the Bayesian analysis.

Phylogenetic reconstruction was carried out on exon 4 nucleotide sequences from a similar set of taxa as in Miller et al. [22]. In this previous study, bootstrap values supporting many of the class I gene clusters among vertebrates were low, as is the case in our study when the *Amcr-UA* and iguanine *UB* fragments are included (Figure 4). However, there is strong nodal support for the monophyly of all squamate class I sequences (*A. cristatus*, *C. subcristatus*, *I. iguana*, *A. ameiva*, and *N. sipedon*) included in the analysis, suggesting that these genes derive from a common ancestral locus whose descendant lineages have not been identified in any other vertebrate group including *Sphenodon*, which is also a member of the Lepidosauria superorder. However, within squamates, there is moderate support for the basal position of *Amcr-UA* relative to all other published class I sequences.

### Polymorphism of *Amcr-UA* in *Amblyrhynchus cristatus* populations

The majority of exon 3 (248 bp) and the first 235 bp of intron 3 were amplified from 65 marine iguanas from three separate islands representing distinct parts of the Galápagos archipelago. Direct sequencing of PCR products for 62 of these individuals generated a single sequence with no double peaks, which is identical to the full-length genomic fragment. Due to the limited polymorphism exhibited at the population level, these data are not displayed graphically. But it is worth noting that two individuals possess a double peak at a single nucleotide position in exon 3, where one base matches the main *Amcr-UA* type and the other results in a nonsynonymous amino acid change (from threonine to serine at amino acid position 133). Another individual possesses double peaks at two nucleotide positions, one site in exon 3 and another in intron 3; here also, the change in the exon is nonsynonymous (from isoleucine to valine at amino acid position 168)

### Patterns of variation between Iguana species

Primers designed on the *Amcr*-*UA* sequence successfully amplified the majority of exon 3 (248 bp) and the entire intron 3 (∼1,030 bp) of six other species from the Iguaninae subfamily, including a representative of *Conolophus* (Galápagos land iguana), which is thought to be the sister genus of the monospecific *Amblyhrynchus* [39], [40]. No stop codons were found in the amino acid sequence of any of the species examined. Uncorrected pairwise genetic distance (*p-*distance) between species was calculated separately for the exon and intron (Table 3). With the exception of *Conolophus* and *Ctenosaura clarki*, the *p*-distance was lower in exon 3 than in intron 3 for all pairwise comparisons, even though the α-2 domain is known to be polymorphic and under strong diversifying selection in classical class I genes [20].

**Table 3:** Uncorrected pairwise distances (*p-*distance) between *Amcr-UA* and similar sequences from other iguanine species.

	1	2	3	4	5	6	7
**1** *Ctde*		0.04435	0.03226	0.02823	0.03226	0.04435	0.04839
**2** *Ctcl*	0.04546		0.03629	0.03226	0.03629	*0.06048*	0.05645
**3** *Cyco*	0.04789	0.05722		0.00403	0.00806	0.03226	0.02823
**4** *Cyca*	0.04693	0.05822	0.01975		0.00403	0.02823	0.02419
**5** *Cyri*	0.04791	0.05430	0.02073	0.00988		0.03226	0.02823
**6** *Cosu*	0.04671	*0.05694*	0.03846	0.03552	0.03850		0.01210
**7** *Amcr-UA*	0.05366	0.06577	0.04924	0.04633	0.05120	0.02650	

Distances were calculated separately for exon 3 (above the diagonal) and intron 3 (below the diagonal). See methods for Iguaninae taxa abbreviations. Pairwise values between *Ctcl* and *Cosu* are italicized because they deviate from the otherwise typical pattern of higher divergence in intron 3 versus exon 3.

**Figure 5:**
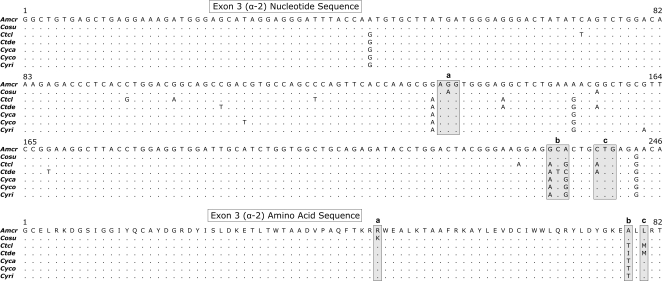
(a) Nucleotide and (b) amino-acid alignments of iguanine exon 3 (α-2 domain) sequences. The lower-case letters and shaded boxes represent specific codon positions that show variation in amino acid residues between species. Species abbreviations are described in the Materials and methods section.

There were 19 polymorphic nucleotide positions in the alignment of iguanine *UA* exon 3 sequences, but only five of these variable sites contributed to nonsynonymous differences between one or more species (Figure 5). As a result, only three of the 82 amino acid positions were polymorphic. While the low divergence of exon 3 compared to intron 3 suggests that the structure of this gene may be conserved, there was insufficient exon variation to carry out *d*_N_/*d*_S_-based analyses as a means of detecting purifying selection acting on this locus.

## Discussion

The basic structure of class I loci is highly conserved across vertebrates. Typically, a single gene encodes a large α chain that contains separate exons for the two membrane-distal peptide-binding domains (α-1 and α-2), a third exon for the membrane-proximal Ig-like domain (α-3), and a number of additional, smaller exons that are responsible for the transmembrane (Tm) and cytoplasmic (Cyt) domains [1]. The Tm/Cyt domains are essential for the standard antigen presentation function of class I molecules because they anchor the receptor structure into the cell membrane of altered self-cells where they can be recognized by CD8^+^ T_C_ cells [1], [2]. However, in the *Amcr-UA* cDNA sequence, the Tm/Cyt domains are largely missing, except for an 11 amino acid extension of the α-3 domain that is followed by a stop codon. The first three residues of this extension are identical to the start of the Tm domain identified in iguanine *UB* and Ameiva lizard class I sequences, but the other eight residues show no apparent match between these sequences.

Interestingly, the genomic data did not reveal the presence of a Tm/Cyt coding region in between the unexpected early stop codon and the downstream 3′ UTR sequence, and there is no evidence of a cryptic, hidden, or vestigial splice site in the 3′ end of the gene. Therefore, the lack of these domains in the cDNA is not likely to be a result of incomplete transcription or a splicing event. Rather, it seems apparent that the Tm/Cyt region is simply absent at this locus. One possible explanation for the match between the *Amcr-UA* α-3 extension and the start of the iguanine *UB* and Ameiva lizard Tm sequences (the “EEP” motif) is that the ancestor of *Amcr-UA* underwent a genomic rearrangement in which a small portion of the Tm-containing exon was transferred to the end of the α-3 encoding exon. Characterization and comparison of similar genes across other squamates may reveal the pattern and timing of such a modification. In particular, it would be interesting to obtain full-length sequences of *Amcr-UA* orthologues from the other closely related iguana species used in this study to determine whether they possess the same truncation at the 3′ end of the CDS.

However, it is also conceivable that intact exons coding for Tm/Cyt domains do exist downstream of the available genomic sequence and simply are not transcribed, at least not at easily detectable levels in the blood. Therefore, future work should involve the use of large insert vectors to explore adjacent stretches of genomic DNA in order to rule out this possibility. In addition, it is equally important to measure expression levels of Amcr-UA in different tissues, and perform a more exhaustive search for a full-length version of this molecule containing a membrane-spanning domain.

Despite the apparent lack of Tm/Cyt domains, which are necessary for classical MHC antigen presentation, the results presented here suggest that the *Amcr*-*UA* sequence represents a functional class I gene and not a pseudogene. The presence of the gene in the cDNA pool shows that it is expressed at some level in the blood. In addition, both the genomic and cDNA sequences do not contain any additional stop codons in the CDS, as might be found in a pseudogene. There are also no stop codons in any of the exon 3 *UA* sequences from other iguanine species.

The presence of a polyadenylation signal in the 3′ UTR is essential for the transcription of a functional gene. While neither of the canonical polyadenylation signals (AATAAA or ATTAAA) were found in the *Amcr-UA* cDNA sequence, several alternative sites were identified, including ATAAA, which is known from fish [41], and AAGAAA, from humans [31]. Moreover, Beaudoing et al. [30] showed that the signal sequence and its position from the poly(A) region vary greatly within the human genome.

The higher divergence in intron 3 compared to exon 3 among the iguanine *UA* sequences suggests that this gene is conserved and under some degree of purifying selection, or at least is not evolving neutrally as would be expected for a nonfunctional pseudogene. However, other physiological and molecular data, including expression at the protein level, must be collected in order to confirm the functionality of *Amcr-UA*. But for the purpose of discussion, we will proceed with the assumption that this gene is functional in order to explore its characteristics and relationship with other vertebrate class I genes.

Despite the absence of a Tm/Cyt region, the *Amcr-UA* sequence shares many of the conserved peptide-binding residues that are suggestive of classical class I function (Figure 3). However, the comparison of *UA* exon 3 sequences between Iguaninae taxa shows that there is little adaptive divergence in the antigen-binding region between species, suggesting that this gene is under purifying selection, rather than balancing-selection, and has a conserved function. Thus, it is possible that the product of this gene is involved in antigen binding, but not in the classical sense.

Although Kaufman et al. [24] interpreted the presence of certain conserved residues at peptide-binding sites as preliminary evidence of classical function, many of these amino acid character states are maintained in nonclassical genes such as the human HLA-E, -F, and -G loci, as well as the mouse H2-M3 gene. None of these loci differ from the classical mammalian type by more than three out of the nine residues [36]. Thus, it is not at all unprecedented that *Amcr-UA* displays several nonclassical features while still showing signs of peptide-binding ability.

Since a lack of polymorphism is an important criterion for describing nonclassical loci, sequence data was collected from exon 3 and intron 3 of *Amcr-UA* for three marine iguana populations. The near absence of exon 3 variability in these samples seems to support the pattern of purifying selection indicating a conserved function. However, the simultaneous lack of diversity in the adjacent intron indicates either strong linkage to the conserved exon or perhaps that an insufficient amount of time has passed for a large number of substitutions to accumulate in the intron. The latter pattern would not be surprising since mitochondrial DNA evidence suggests that marine iguana populations are not genetically diverse and may have recently expanded in the Galápagos archipelago [42]. Therefore, the unclear evolutionary history of this species makes it difficult to attribute the dearth of polymorphism in *Amcr-UA* to its nonclassical function.

While rare, several truncated class I molecules are known to lack Tm/Cyt regions in humans and mice. For example, the mouse Q10 gene contains a 13 bp deletion in the exon encoding the Tm domain, causing a frame shift and the introduction of a premature termination signal downstream in the same exon. In addition, the remaining transcribed portion of the Tm domain has numerous polar amino acid residues that would likely prevent its insertion into the cell membrane. Therefore, it seems clear that the Q10 molecule is not involved in classical antigen presentation. It also possesses several other nonclassical features, including a lack of polymorphism and expression that is almost exclusive to mouse liver cells [43]–[45]. Several studies have shown that Q10 is likely secreted and can bind a wide array of non-self peptides in a similar manner to classical molecules, but its function is still not well understood [44], [46]. While there is no phylogenetic similarity between *Amcr-UA* and the mouse Q10, the characteristics of the latter gene demonstrate that truncated, soluble, secreted class I molecules can exist which lack genetic variation and maintain protein binding capabilities.

Numerous other classical and nonclassical class I molecules are known to exist in soluble form for secretion. For example, the human HLA-G locus possesses all of the coding features of a membrane-bound class I receptor, but is sometimes subjected to alternative transcription where the Tm/Cyt domains are deleted. This molecule is expressed specifically in placental tissue and is secreted during pregnancy [47]. The role of this gene is also not well understood, but is thought to be involved in inducing apoptosis in activated maternal CD8^+^ T cells [48]. Classical human class I genes (HLA-A, -B, and -C) are also known to exist in soluble forms and to play a role in cell death of activated T cells [49]. Nevertheless, a unique feature of the *Amcr-UA* sequence which distinguishes it from the Q10 and HLA-G loci, as well as other soluble class I molecules, is that there does not appear be any sign of a Tm/Cyt coding sequence in the genomic DNA, regardless of whether it is transcribed or not; but again, additional support for this conclusion must come from further collection of sequence data in the 3′ region of the gene.

Some truncated class Ib molecules, such as those in the mouse Qa-2 family, are not necessarily secreted, but are rather linked to the cell membrane through a glycosylphosphatidylinositol (GPI) anchor that is added to the carboxyl terminus of the protein during posttranslational modification [50], [51]. Therefore, the apparent lack of a membrane-spanning domain in *Amcr-UA* doesn't rule out its expression on the cell surface or its involvement in the primary T cell response.

Overall, the *Amcr-UA* CDS showed the highest similarity with iguanine *UB* and Ameiva lizard sequences. In addition, phylogenetic reconstruction supported the monophyly of all available squamate class I sequences. The basal position of *Amcr-UA* relative to all other squamate class I sequences suggests that this gene diverged very early in the evolution of this reptilian order. The most recent common ancestor of iguanines and the two groups represented by the Ameiva lizard (Family: Teiidae) and northern water snake (Suborder: Serpentes) is estimated to have existed between 179–206 million years ago [14], providing a minimum time for the split of *Amcr-UA* from class Ia genes in squamates. Outside of the squamate grouping, the tree topology is not well supported, suggesting that class I sequences are highly divergent among vertebrate groups.

In summary, the *Amcr-UA* sequence possesses several characteristics of a functional, non-classical class I gene with a conserved protein structure. Additional work must be conducted to understand whether it is expressed at the level of the protein and what its position is in the genome relative to published classical loci [16]. While *Amcr-UA* is most closely related to the other published squamate sequences, it does not cluster with class Ia sequences from the same species, suggesting that it has long been on a separate evolutionary trajectory. Further characterization of *UA*-like sequences from other squamates will reveal whether this gene is part of a lineage that has maintained a non-classical function over the course of squamate evolution.

## References

[1] Klein J (1986). Natural history of the major histocompatibility complex..

[2] Ploegh H, Watts C (1998). Antigen recognition.. Curr Opin Immunol.

[3] Bjorkman PJ, Parham P (1990). Structure, function, and diversity of class I major histocompatibility complex molecules.. Annu Rev Biochem.

[4] Potts WK, Wakeland EK (1990). Evolution of diversity at the major histocompatibility complex.. Trends Ecol Evol.

[5] Brigl M, Brenner MB (2004). CD1: Antigen presentation and T cell function.. Annual Rev Immunol.

[6] Braud VM, Allan DS, McMichael AJ (1999). Functions of nonclassical MHC and non-MHC-encoded class I molecules.. Curr Opin Immunol.

[7] Hansen TH, Huang S, Arnold PL, Fremont DH (2007). Patterns of nonclassical MHC antigen presentation.. Nat Immunol.

[8] Hughes AL, Nei M (1989). Evolution of the major histocompatibility complex: independent origin of nonclassical class I genes in different groups of mammals.. Mol Biol Evol.

[9] Nei M, Gu X, Sitnikova T (1997). Evolution by the birth-and-death process in multigene families of the vertebrate immune system.. Proc Natl Acad Sci USA.

[10] Rada C, Lorenzi R, Powis SJ, Vandenbogaerde J, Parham P (1990). Concerted evolution of class I genes in the major histocompatibility complex of murine rodents.. Proc Natl Acad Sci USA.

[11] Hess CM, Edwards SV (2002). The evolution of the major histocompatibility complex in birds.. Bioscience.

[12] Belov K, Deakin JE, Papenfuss AT, Baker ML, Melman SD (2006). Reconstructing an ancestral mammalian immune supercomplex from a marsupial major histocompatibility complex.. PLoS Biol.

[13] Rest JS, Ast JC, Austin CC, Waddell PJ, Tibbetts EA (2003). Molecular systematics of primary reptilian lineages and the tuatara mitochondrial genome.. Mol Phylogenet Evol.

[14] Vidal N, Hedges SB (2005). The phylogeny of squamate reptiles (lizards, snakes, and amphisbaenians) inferred from nine nuclear protein-coding genes.. C R Biologies.

[15] Vitt LJ, Pianka ER, Cooper WE, Schwenk K (2003). History and the global ecology of squamate reptiles.. Am Nat.

[16] Glaberman S, Caccone A (2008). Species-specific evolution of class I MHC genes in iguanas (Order: Squamata; Subfamily: Iguaninae).. Immunogenetics.

[17] Flajnik M (2004). Comparative genomics of the MHC.. Tissue Antigens.

[18] Radtkey RR, Becker B, Miller RD, Riblet R, Case TJ (1996). Variation and evolution of class I Mhc in sexual and parthenogenetic geckos.. Proc R Soc London Ser B.

[19] Sambrook J, Russell DW (2001). Molecular cloning : a laboratory manual..

[20] Hughes AL, Yeager M (1998). Natural selection at major histocompatibility complex loci of vertebrates.. Annu Rev Genet.

[21] Edgar RC (2004). MUSCLE: multiple sequence alignment with high accuracy and high throughput.. Nucleic Acids Res.

[22] Miller HC, Belov K, Daugherty CH (2006). Proceedings of the SMBE tri-national young investigators' workshop 2005. MHC class I genes in the tuatara (*Sphenodon* spp.): evolution of the MHC in an ancient reptilian order.. Mol Biol Evol.

[23] Tamura K, Dudley J, Nei M, Kumar S (2007). MEGA4: Molecular evolutionary genetics analysis (MEGA) software version 4.0.. Mol Biol Evol.

[24] Kaufman J, Salomonsen J, Flajnik M (1994). Evolutionary conservation of MHC class I and class II molecules–different yet the same.. Semin Immunol.

[25] Nylander JAA (2004). MrModeltest v2. Evolutionary Biology Centre, Uppsala University: Program distributed by the author.

[26] Posada D, Crandall KA (1998). MODELTEST: testing the model of DNA substitution.. Bioinformatics.

[27] Ronquist F, Huelsenbeck JP (2003). MrBayes 3: Bayesian phylogenetic inference under mixed models.. Bioinformatics.

[28] Jobb G, von Haeseler A, Strimmer K (2004). TREEFINDER: a powerful graphical analysis environment for molecular phylogenetics.. BMC Evol Biol.

[29] Klein J, Bontrop RE, Dawkins RL, Erlich HA, Gyllensten UB (1990). Nomenclature for the major histocompatibility complexes of different species - a proposal.. Immunogenetics.

[30] Beaudoing E, Freier S, Wyatt JR, Claverie JM, Gautheret D (2000). Patterns of variant polyadenylation signal usage in human genes.. Genome Res.

[31] Plant MH, Laneuville O (1999). Characterization of a novel transcript of prostaglandin endoperoxide H synthase 1 with a tissue-specific profile of expression.. Biochem J.

[32] Pauws E, van Kampen AH, van de Graaf SA, de Vijlder JJ, Ris-Stalpers C (2001). Heterogeneity in polyadenylation cleavage sites in mammalian mRNA sequences: implications for SAGE analysis.. Nucleic Acids Res.

[33] Tian B, Hu J, Zhang HB, Lutz CS (2005). A large-scale analysis of mRNA polyadenylation of human and mouse genes.. Nucleic Acids Res.

[34] Adhikary G, Gupta S, Sil P, Saad Y, Sen S (2005). Characterization and functional significance of myotrophin: A gene with multiple transcripts.. Gene.

[35] Madden DR (1995). The three-dimensional structure of peptide-MHC complexes.. Annu Rev Immunol.

[36] Shum BP, Rajalingam R, Magor KE, Azumi K, Carr WH (1999). A divergent non-classical class I gene conserved in salmonids.. Immunogenetics.

[37] Grimholt U, Hordvik I, Fosse VM, Olsaker I, Endresen C (1993). Molecular cloning of major histocompatibility complex class I cDNAs from Atlantic salmon (*Salmo salar*).. Immunogenetics.

[38] Timon M, Elgar G, Habu S, Okumura K, Beverley PC (1998). Molecular cloning of major histocompatibility complex class I cDNAs from the pufferfish *Fugu rubripes*.. Immunogenetics.

[39] Rassmann K (1997). Evolutionary age of the Galápagos iguanas predates the age of the present Galápagos Islands.. Mol Phylogenet and Evol.

[40] Wiens JJ, Hollingsworth BD (2000). War of the iguanas: Conflicting molecular and morphological phylogenies and long-branch attraction in iguanid lizards.. Syst Biol.

[41] Kwon JY, Prat F, Randall C, Tyler CR (2001). Molecular characterization of putative yolk processing enzymes and their expression during oogenesis and embryogenesis in rainbow trout (*Oncorhynchus mykiss*).. Biol Reprod.

[42] Rassmann K, Tautz D, Trillmich F, Gliddon C (1997). The microevolution of the Galápagos marine iguana *Amblyrhynchus cristatus* assessed by nuclear and mitochondrial genetic analyses.. Mol Ecol.

[43] Cosman D, Kress M, Khoury G, Jay G (1982). Tissue-specific expression of an unusual H-2 (class I)-related gene.. Proc Natl Acad Sci USA.

[44] Kress M, Cosman D, Khoury G, Jay G (1983). Secretion of a transplantation-related antigen.. Cell.

[45] Mellor AL, Weiss EH, Kress M, Jay G, Flavell RA (1984). A nonpolymorphic class I gene in the murine major histocompatibility complex.. Cell.

[46] Zappacosta F, Tabaczewski P, Parker KC, Coligan JE, Stroynowski I (2000). The murine liver-specific nonclassical MHC class I molecule Q10 binds a classical peptide repertoire.. J Immun.

[47] Rebmann V, Pfeiffer K, Passler M, Ferrone S, Maier S (1999). Detection of soluble HLA-G molecules in plasma and amniotic fluid.. Tissue Antigens.

[48] Fournel S, Aguerre-Girr M, Huc X, Lenfant F, Alam A (2000). Soluble HLA-G1 triggers CD95/CD95 ligand-mediated apoptosis in activated CD8(+) cells by interacting with CD8.. J Immunol.

[49] Contini P, Ghio M, Poggi A, Filaci G, Indiveri F (2003). Soluble HLA-A,-B,-C and -G molecules induce apoptosis in T and NKCD8(+) cells and inhibit cytotoxic T cell activity through CD8 ligation.. Eur J Immunol.

[50] Stiernberg J, Low MG, Flaherty L, Kincade PW (1987). Removal of Lymphocyte Surface Molecules with Phosphatidylinositol-Specific Phospholipase-C - Effects on Mitogen Responses and Evidence That Thb and Certain Qa-Antigens Are Membrane-Anchored Via Phosphatidylinositol.. J Immunol.

[51] Stroynowski I, Tabaczewski P (1996). Multiple products of class Ib Qa-2 genes which ones are functional?. Res Immunol.

[52] Koller BH, Orr HT (1985). Cloning and complete sequence of an HLA-A2 gene: analysis of two HLA-A alleles at the nucleotide level.. J Immunol.

